# CT-Assessment of Epicardial Fat Identifies Increased Inflammation at the Level of the Left Coronary Circulation in Patients with Atrial Fibrillation

**DOI:** 10.3390/jcm13051307

**Published:** 2024-02-26

**Authors:** Renáta Gerculy, Imre Benedek, István Kovács, Nóra Rat, Vasile Bogdan Halațiu, Ioana Rodean, Lehel Bordi, Emanuel Blîndu, Aurelian Roșca, Botond-Barna Mátyás, Evelin Szabó, Zsolt Parajkó, Theodora Benedek

**Affiliations:** 1 Clinic of Cardiology, Mures, County Emergency Clinical Hospital, 540142 Târgu Mures, Romania; gerculy_renata@yahoo.com (R.G.); imre.benedek@umfst.ro (I.B.); istvan.kovacs@umfst.ro (I.K.); ioana.rodean@umfst.ro (I.R.); laszlo-lehel.bordi@umfst.ro (L.B.); emi.blindu@yahoo.com (E.B.); rosca_aurelian@yahoo.com (A.R.); matyas_botond@yahoo.com (B.-B.M.); szaboevelin22@yahoo.com (E.S.); p_zsolt92@yahoo.com (Z.P.); theodora.benedek@umfst.ro (T.B.); 2 Center of Advanced Research in Multimodality Cardiac Imaging, CardioMed Medical Center, 540124 Târgu Mures, Romania; 3 Doctoral School of Medicine and Pharmacy, University of Medicine, Pharmacy, Science and Technology “George Emil Palade” of Târgu Mures, 540139 Târgu Mures, Romania

**Keywords:** perivascular inflammation, atrial fibrillation, fat attenuation index, coronary computed tomography angiography

## Abstract

**Background:** Atrial fibrillation (AF) can often be triggered by an inflammatory substrate. Perivascular inflammation may be assessed nowadays using coronary computed tomography angiography (CCTA) imaging. The new pericoronary fat attenuation index (FAI HU) and the FAI Score have prognostic value for predicting future cardiovascular events. Our purpose was to investigate the correlation between pericoronary fat inflammation and the presence of AF among patients with coronary artery disease. **Patients and methods:** Eighty-one patients (mean age 64.75 ± 7.84 years) who underwent 128-slice CCTA were included in this study and divided into two groups: group 1 comprised thirty-six patients with documented AF and group 2 comprised forty-five patients without a known history of AF. **Results:** There were no significant differences in the absolute value of fat attenuation between the study groups (*p* > 0.05). However, the mean FAI Score was significantly higher in patients with AF (15.53 ± 10.29 vs. 11.09 ± 6.70, *p* < 0.05). Regional analysis of coronary inflammation indicated a higher level of this process, especially at the level of the left anterior descending artery (13.17 ± 7.91 in group 1 vs. 8.80 ± 4.75 in group 2, *p* = 0.008). **Conclusions:** Patients with AF present a higher level of perivascular inflammation, especially in the region of the left coronary circulation, and this seems to be associated with a higher risk of AF development.

## 1. Introduction

### Atrial Fibrillation and Pericoronary Inflammation

Atrial fibrillation (AF) stands as the most prevalent arrhythmia encountered in clinical practice. Its frequency has been steadily rising over the past few decades, primarily attributed to the aging population and enhanced survival rates of patients with other cardiovascular conditions. In 2016, the Global Burden of Disease project estimated that approximately 46.3 million individuals worldwide were affected by AF [1]. The incidence and prevalence of AF have been on the rise over the last 20 years and are expected to continue increasing over the next 30 years, particularly in countries with middle socio-demographic indices. This trend poses one of the largest epidemics and public health challenges globally [2]. The increasing prevalence of AF constitutes a substantial health challenge, linked to notable morbidity and mortality, along with escalated healthcare expenses. AF is a complex and varied arrhythmia that manifests in diverse clinical contexts [3]. AF prediction remains a significant challenge worldwide. Artificial intelligence (AI)-enhanced algorithms implementing machine learning (ML) and deep learning (DL) techniques can improve the accuracy of prediction, overcoming the conventional statistical analysis [4]. Quantifying atrial fibrosis using cardiac magnetic resonance with DL techniques is a new challenging method for AF prediction [5]. Conventional statistical methods are based on patient electrocardiography patterns, demographic data, comorbidities, and mostly other risk factors. Nevertheless, challenges and limitations persist with clinical risk scores, constraining their applicability to specific populations [6].

Over the course of the last few years, an increasing body of evidence has indicated a strong association between increased systemic inflammation and AF [7,8]. Inflammatory processes significantly impact both the electrophysiology and structural properties of the atria [9]. Extensive scientific research has provided compelling evidence linking chronic inflammation to the initiation, advancement, and destabilization of atherosclerosis. Atherosclerosis, a chronic inflammatory disease, has the potential to contribute to the promotion of AF. Inflammation and oxidative stress are among the main mechanisms contributing to endothelial dysfunction and arterial damage, promoting the onset of AF [10].

Recent studies have demonstrated that pericoronary adipose tissue (PCAT) undergoes alterations in its biology and structure in response to vascular inflammation [11]. PCAT surrounding the coronary arteries is in a reciprocal interaction with the vascular wall, which releases chemokines that induce changes in the surrounding perivascular space [12,13].

These modifications can occur prior to the appearance of any atherosclerotic plaques within the coronary artery itself and lead to corresponding shifts in the PCAT phenotype, which is detectable by coronary computed tomography angiography (CCTA). The increase in the water-to-lipid ratio with consequent reduction in adipocyte size in proximity to inflamed arteries are features easily detectable by CCTA [14]. The PCAT attenuation index or fat attenuation index (PCAT-FAI) is a promising CCTA imaging biomarker of coronary vascular inflammation with a high prognostic value for future cardiovascular events [15,16].

An increasing body of evidence suggests a strong link between inflammation in coronary arteries and ischemic heart disease, but the connection between PCAT inflammation and non-ischemic heart diseases like AF remains unclear. While PCAT changes are generally associated with atherosclerosis and ischemic heart disease, their influence on AF is not well defined and warrants further exploration.

The aim of our study was to investigate the correlation between pericoronary fat inflammation and the presence of atrial fibrillation among patients with coronary artery disease. We also compared the CA-Ri Heart Risk derived from FAI Score values, the plaque burden, and other clinical risk factors in patients with AF versus those in sinus rhythm.

## 2. Materials and Methods

### 2.1. Study Population

This retrospective cohort study included 81 individuals with low to intermediate risk of coronary artery disease (CAD), as indicated by initial symptoms of chest pain. The participants were categorized into two groups: the first group comprised 36 patients with a confirmed history of AF, either presently or in past medical records, while the second group consisted of 45 patients of similar age and gender with no known history of AF.

### 2.2. Study Procedures

All study participants underwent CCTA using a 128-slice CT scanner (Siemens Somatom Definition AS, Siemens Healthcare, Erlangen, Germany) to evaluate coronary anatomy and detect coronary plaques. The scan was gated with a heart rate below 70 beats per minute, and the parameters included a tube voltage of 120 kV, gantry rotation time of 0.33 s, and a collimation of 128 × 0.6. Beta-blockers were administered to patients with a resting heart rate above 70 beats per minute, and vital parameters were monitored during the administration of intravenous or oral beta-blockers. The acquisition process began with a native scan for coronary calcium assessment, followed by the administration of 80–100 mL of iodine-based contrast material based on body weight, with a 50 mL saline chase at a flow rate of 5.5–6 mL/s during inspiratory breath-hold. The scans obtained were stored in a specialized electronic imaging database for later offline post-processing. Subsequently, these images were sent to Caristo Diagnostics Center in Oxford, U.K., for the measurement of PCAT-FAI. Following the analysis, the results were sent back to our center and saved in an electronic database for additional analysis.

In addition to the CCTA images, we recorded the coronary calcium score (CCS), as well as the degree of stenosis in each coronary artery (quantified as a stenosis percentage ranging from 0 to 100%). We also collected demographic data, information on comorbidities, laboratory findings, and echocardiography measurements for each patient. Subsequent statistical analysis was performed using GraphPad Prism 10.1.0 software, and results were considered statistically significant if the two-sided *p*-value was 0.05 or less.

We evaluated two distinct parameters by assessing PCAT characteristics. Firstly, the FAI-HU, which is a graphical depiction of pericoronary inflammation levels without adjustments represented in Hounsfield Units (HUs). Secondly, the FAI Score, which is a personalized assessment measuring coronary inflammation in the three major epicardial coronary arteries adjusted by age and gender. Finally, we compared the CaRi-Heart^®^ Risk, a tool for evaluating an individual’s risk of a fatal cardiac event over eight years, which incorporates the FAI Score into a prognostic model that includes risk factors like diabetes, smoking, hyperlipidemia, and hypertension, thus providing a comprehensive view of atherosclerotic plaque burden and patient risk.

CCTA-derived advanced techniques allowed us to quantify attenuation gradients originating from the outer surface of the vascular wall, the place that undergoes changes due to inflammatory signals from the vascular wall, resulting in perivascular edema. The attenuation of adipose tissue in CCTA typically falls within the more negative range (closer to −190 HUs). However, in the presence of smaller adipocytes with lower lipid content, the attenuation shifts toward a less negative range (closer to −30 HUs). This shift signifies a shift in tissue composition from lipid-rich to a higher proportion of the aqueous phase, indicating the measurement of inflammation throughout the vascular wall.

### 2.3. Statistical Analysis

After quantifying the PVAT-FAI for each coronary artery, the collected data were transmitted to our center and stored in an electronic Microsoft Excel database (Microsoft Office 2019). Statistical analysis was conducted using GraphPad Prism 10 software (GraphPad Software, Inc., San Diego, CA, USA). This study encompassed a PCAT-FAI analysis of 81 coronary arteries: 81 on the left anterior descending artery (LAD), 81 on the circumflex artery (LCX), and 81 on the right coronary artery (RCA). Categorical variables (nominal) were presented as integer values (percentages) and compared between groups using the Chi-square test (χ^2^) and its corresponding variables. Numeric data were expressed as mean ± standard deviation, and either a Mann–Whitney or an unpaired Student’s *t*-test was applied. Pearson correlation analysis was conducted to assess correlations between the PVAT-FAI and other variables as appropriate. Results were deemed statistically significant if the two-sided *p*-value was 0.05.

## 3. Results

### 3.1. Baseline Characteristics

**Figure 1:**
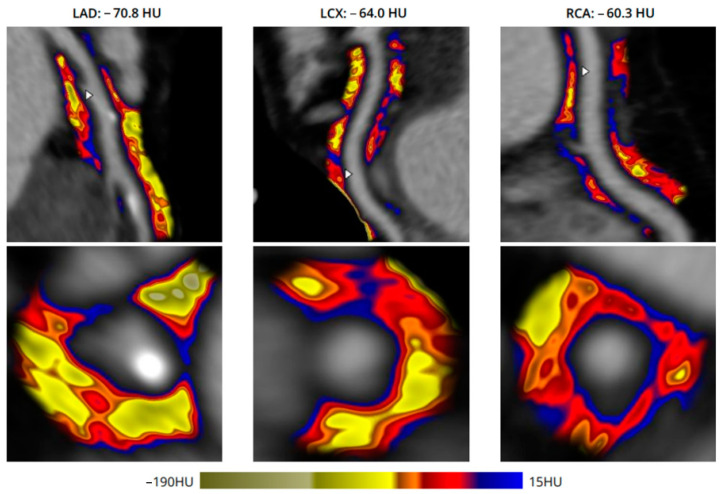
An example colored mapping representation of an abnormal FAI from the three major coronary arteries. Yellow arrows demonstrate stable atherosclerotic lesions with low FAI values, while blue arrows mean higher FAI values and higher inflammation. LAD: left anterior descending artery; LCX: left circumflex artery; RCA: right coronary artery; HU: Hounsfield Unit.

The mean age at the time of the scan in the two study groups did not show a significant difference (64.75 ± 7.84 vs. 62.58 ± 7.89, *p* = ns) (Table 1). The gender distribution in the two study groups was quite similar, with 25 (69.44%) vs. 33 (71.74%) represented by males, indicating a relatively balanced distribution within both groups. There were no statistically significant differences within the study groups regarding the mean body mass index (25.33 ± 5.29 vs. 27.85 ± 3.59, *p* = ns), hypertension (72.22% vs. 71.43%, *p* = ns), hypercholesterolemia (41.67% vs. 52.17%, *p* = ns), diabetes (30.56% vs. 23.91%, *p* = ns), or smoking habits (5.56% vs. 19.57%, *p* = ns). Left atrium size was significantly higher among patients with atrial fibrillation compared with those without atrial fibrillation (45.10 ± 7.18 vs. 39.20 ± 6.26, *p* = 0.0011) (Table 1). Figure 1 demonstrates the case of a single patient who underwent CCTA and was referred to the partner center for processing the images utilizing AI algorithms.

### 3.2. Laboratory Parameters 

The results of the laboratory analyses are presented in Table 2. There were no significant differences between the study groups regarding creatinine level (1.02 ± 0.28 vs. 0.96 ± 0.29, *p* = ns), hemoglobin (14.36 ± 1.59 vs. 14.79 ± 1.07, *p* = ns), leucocyte count (7880 ± 2125 vs. 8153 ± 2303, *p* = ns), total cholesterol (166 ± 33.77 vs. 174.6 ± 59.45, *p* = ns), HDL-cholesterol (45.12 ± 12.23 vs. 45.11 ± 7.83, *p* = ns), LDL-cholesterol (88.54 ± 22.75 vs. 99.1 ± 42.6, *p* = ns), triglycerides (127.2 ± 72.65 vs. 120.7 ± 60.4, *p* = ns), or uric acid (5.48 ± 1.66 vs. 5.75 ± 0.77, *p* = ns).

### 3.3. CCTA Standard Evaluation of the Coronary Arteries

**Figure 2:**
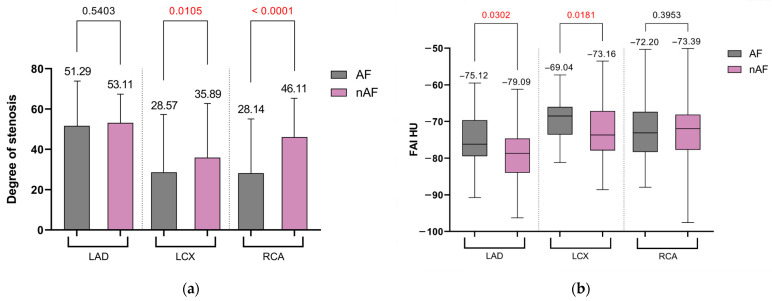
The degree of stenosis (%) at the level of the three coronary arteries (**a**). Regional analysis of FAI (HU) at the level of the three main coronary arteries (**b**). Significant *p* values are marked as red.

The severity of stenosis in the left anterior descending coronary artery (LAD) was comparable between both groups (53.11% vs. 51.29%, *p* = 0.5403). However, the left circumflex artery (LCX) and right coronary artery (RCA) showed significantly greater lesion severity in patients without AF history (LCX: 35.89% vs. 28.57%, *p* = 0.0105; RCA: 46.11% vs. 28.14%, *p* < 0.0001), as shown in Figure 2a. 

### 3.4. Coronary Inflammation and Atrial Fibrillation

In terms of conventional FAI-HU measurements at the LAD (−79.09 vs. −75.12, *p* = 0.0302) and LCX (−73.16 vs. −69.04, *p* = 0.0181), the FAI shifted toward a less negative range, indicating the presence of smaller adipocytes with lower lipid content, a marker of increased inflammation at the group of patients with atrial fibrillation. This trend was not significant at the RCA site (*p* = 0.3953) (Figure 2b). Regional analysis of coronary inflammation indicated higher levels of inflammation, especially at the level of the left anterior descending artery (13.17 ± 7.91 in group 1 vs. 8.80 ± 4.75 in group 2, *p* = 0.008) in patients with AF. 

**Figure 3:**
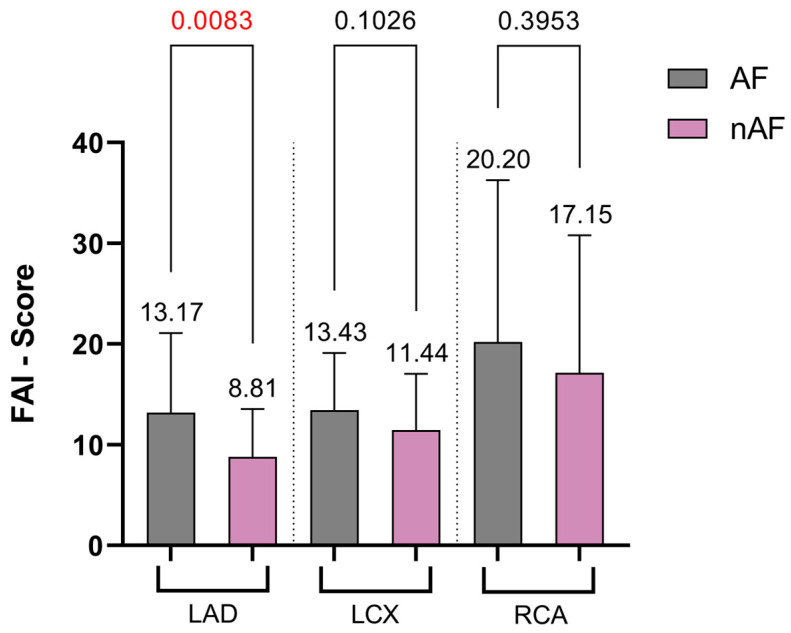
Regional analysis of FAI Score values between the two study groups. Significant *p* values are marked as red.

**Figure 4:**
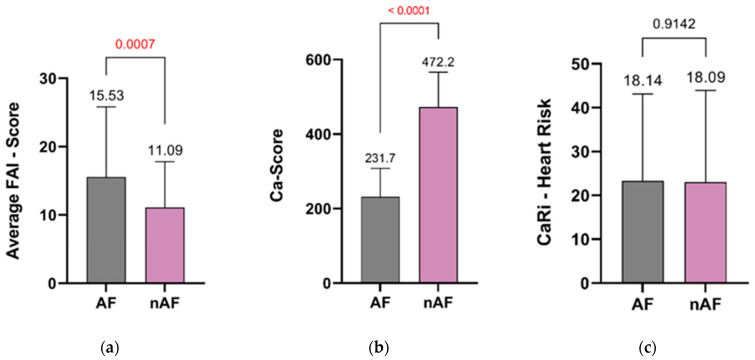
Average FAI score (**a**); coronary calcium score (**b**); and CaRi-Heart Risk (**c**). Significant *p* values are marked as red.

At the level of LCX, the FAI Score was apparently higher in patients with AF; however, the difference was not statistically different (13.43 ± 5.69 vs. 11.44 ± 5.6, *p* = ns) neither at the level of right coronary artery (RCA: 20.20 ± 16.08 SD vs. 17.15 ± 13.66 SD, *p* = ns) (Figure 3). The average FAI Score, tailored to reflect coronary inflammation while considering age and gender, was notably higher in AF patients (15.53 ± 10.29) compared with those without AF (11.09 ± 6.70, *p* = 0.0007) (Figure 4). Patients without AF demonstrated a notably higher CCS than those with AF (472.2 vs. 231.7, *p* < 0.0001). The CaRi-Heart Risk score, predicting the 8-year risk of a fatal cardiac event by integrating personalized FAI Scores, plaque burden, and clinical risk factors, was higher in the AF group, although the difference between the groups was not statistically significant (18.14 ± 14.09 vs. 18.09 ± 13.59, *p* = 0.9142), as illustrated in Figure 4. As well, the left atrium size was significantly higher in the group of patients with atrial fibrillation (45.10 vs. 39.20, *p* = 0.011).

## 4. Discussion

The main finding of our study was the demonstration that patients with AF present a regional variation in pericoronary inflammation. The left coronary arteries seem to exhibit a higher level of inflammation in patients with AF, and this may be related to the origin of the arrhythmia from the left heart. This is the first study published in the literature that demonstrates the existence of regional differences between inflammatory status in the left versus right coronary circulations in patients with AF.

At the same time, we demonstrated that (1) in patients with chest pain, those with AF have a higher degree of epicardial fat inflammation, while those without AF have more extensive calcifications in the coronary arteries, and (2) the assessment of epicardial fat inflammation using the novel FAI technology may identify an increased risk for developing atrial fibrillation. 

We found an increased average FAI Score in patients with AF compared with the control lot, which indicates a higher inflammation in the epicardial fat surrounding coronary arteries. We also recorded a higher calcium score in patients without AF, reflecting a more advanced atherosclerosis. 

We also demonstrated that the relationship between vascular inflammation and atrial fibrillation is not correlated with traditional risk factors. In our study population, there were no statistically significant differences between the two study groups observed in baseline characteristics such as age, sex, comorbidities, and common laboratory parameters. Our results highlight the published literature findings indicating that the FAI is independent of other parameters [17]. Furthermore, the severity of coronary stenosis was higher at the level of RCA and LCX in the group with no AF; however, the inflammation represented by FAI was higher among patients with AF independently of the degree of stenosis. 

According to our study, the association between perivascular adipose tissue and AF is suggested by higher values of the FAI Score among patients with AF. The average FAI Score was significantly higher in the presence of AF despite the other investigated parameters. In the meantime, CCS was significantly higher in the group without AF. This finding suggests that the extent of calcification and stenosis degree do not correlate with the severity of coronary artery disease or coronary inflammation or indirectly with the onset of AF. The CaRi-Heart Risk score was marginally higher in the AF group, but the difference was not statistically significant, which also points to the potential utility of FAI and CCTA in evaluating and managing patients with or at risk of AF. 

The significant enlargement of the left atrium in AF patients underscores the well-established association between left atrial size and AF. Although there is some debate about whether a dilated atrium can predict post-ablation AF recurrence, enlargement is a recognized marker of AF and its severity [18,19,20]. The higher CCS in patients without AF and the greater severity of lesions in the LCX and RCA highlight a complex interplay between AF and CAD. This relationship is emphasized by the shared risk factors and common pathophysiological characteristics between AF and CAD, where systemic inflammation plays a crucial role in both conditions [20]. It is also important to note the effectiveness of PCAT-FAI, as determined by CCTA, in detecting inflammation in PCAT [21,22]. This effectiveness is particularly notable given its independence from coronary calcification and its lack of correlation with coronary lumen stenosis [23,24].

This study highlights the association between PCAT and AF, with a higher average FAI Score in AF patients, suggesting increased coronary inflammation. This is consistent with research indicating a strong correlation between CAD and AF, with shared risk factors and inflammatory pathways playing prominent roles in their development [25]. Inflammatory processes have been closely associated with AF, as evidenced by increased levels of inflammatory biomarkers and inflammatory infiltrates in the atrial tissues of AF patients [26]. CAD, often linked with systemic inflammation, is noted as a common factor underlying AF [27]. These findings align with the broader understanding that inflammation is a key factor in heart diseases, including AF. 

This study’s findings suggest a certain role of local factors related to coronary circulation in the development of AF. A potential explanation for this observation may be related to the different hemorheologic patterns of coronary circulation in the two segments (left and right), with a potential role of local shear stress in influencing coronary inflammation. This could open new avenues for the prevention of AF and even for the development of new therapeutic strategies for this condition targeting inflammation.

## 5. Study Limitations and Future Directions

The purpose of this study was to investigate the correlation between pericoronary fat inflammation and the presence of AF among patients with coronary artery disease, using AI-assisted technology integrated into the CaRi-Heart medical technology. This integrates standardized FAI mapping with plaque parameters and clinical risk factors to deliver individualized cardiovascular risk assessments. 

However, our study has a few limitations. Firstly, our study cohort was recruited only from a single center, which may not accurately represent the entire population with AF. Secondly, our study included patients with low or intermediate risk of CAD, which could introduce selection bias and limit generalizability. High-risk patients for CAD, who might exhibit an elevated inflammatory burden were not included in our study. However, this exclusion is in line with the 2020 ESC guidelines, which recommend CCTA in cases with a low to intermediate likelihood of CAD. Additionally, our study did not investigate the role of serum inflammatory biomarkers, which could offer deeper insights into the correlation between perivascular inflammation and AF.

Despite these limitations, our study suggests a significant association between coronary inflammation and the risk of AF. However, in light of the above-mentioned limitations, further research is needed to confirm the clinical significance of the FAI Score in the management of coronary inflammation and to establish a relationship with AF. 

More studies are necessary to achieve a thorough understanding of the link between AF and coronary inflammation. Forthcoming studies will integrate high-risk patients who have undergone CCT and will also use inflammatory biomarkers such as C-reactive protein (CRP) and interleukin-6 (IL-6) in a larger number of patients.

## 6. Conclusions

Patients with AF seem to present a higher level of inflammation in the epicardial fat surrounding coronary arteries, especially at the level of the left coronary circulation. A high inflammation at this level, detectable by CCTA using CaRi-Heart technology, may be associated with a higher risk of AF.

## Figures and Tables

**Table 1:** Comparison of demographic data and comorbidities between the two study groups.

Parameters	Group 1 (AF) *n* = 36	Group 2 (nAF) *n* = 45	*p*-Value
Age, mean ± SD	64.75 ± 7.84	62.58 ± 7.89	0.201
Male gender, *n* (%)	25 (69.44)	33 (71.74)	>0.99
Body mass index, mean ± SD	28.33 ± 5.29	27.85 ± 3.59	0.781
Hypertension, *n* (%)	26 (72.22)	40 (71.73)	>0.99
Hypercholesterolemia, *n* (%)	15 (41.67)	24 (52.17)	0.379
Diabetes, *n* (%)	11 (30,56)	11 (23,91)	0.616
Smoking, *n* (%)	2 (5.56)	9 (19.57)	0.101

**Table 2:** Comparison of laboratory analyses between the two study groups.

Parameters	Group 1 (AF) *n* = 36	Group 2 (nAF) *n* = 45	*p*-Value
Creatinine, mean ± SD	1.02 ± 0.28	0.96 ± 0.29	0.462
Urea, mean ± SD	37.91 ± 13.20	35.60 ± 13.20	0.503
Hemoglobin, mean ± SD	14.36 ± 1.59	14.79 ± 1.07	0.312
Leucites, mean ± SD	7880 ± 2125	8153 ± 2303	0.728
Total cholesterol, mean ± SD	166 ± 33.77	174.6 ± 59.45	0.929
HDL cholesterol, mean ± SD	45.12 ± 12.23	41.11 ± 7.83	0.339
LDL cholesterol, mean ± SD	88.54 ± 22.75	91.10 ± 42.60	0.538
Triglycerides, mean ± SD	127.2 ± 72.65	120.7 ± 60.04	0.984
Uric acid, mean ± SD	5.48 ± 1.66	5.75 ± 0.77	0.602

## Data Availability

The data presented in this study are available upon request from the corresponding author. The data are not publicly available due to privacy reasons.
